# Supplementary material for: Identity Leadership, Employee Burnout and the Mediating Role of Team Identification: Evidence from the Global Identity Leadership Development Project
Source: Int J Environ Res Public Health. 2021 Nov 17;18(22):12081. doi: 10.3390/ijerph182212081 (PMC8624344; doi:10.3390/ijerph182212081)
Supplement: Supplementary file 1 [file ijerph-18-12081-s001.zip › ijerph-1425284-supplementary.pdf]

Surveys in all languages

English

Below, we would like you to think about your team and your team leader (i.e. your direct supervisor). Please focus on the same team and the corresponding leader throughout the survey.

My team leader…

	Disagree completely 1	2	3	4	5	6	Agree completely 7
... embodies what the team stands for.	◎	◎	◎	◎	◎	◎	◎
… is representative of members of the team.	◎	◎	◎	◎	◎	◎	◎
... is a model member of the team.	◎	◎	◎	◎	◎	◎	◎
... exemplifies what it means to be a member of the team.	◎	◎	◎	◎	◎	◎	◎
... promotes the interests of members of the team.	◎	◎	◎	◎	◎	◎	◎
... acts as a champion for the team.	◎	◎	◎	◎	◎	◎	◎
... stands up for the team.	◎	◎	◎	◎	◎	◎	◎
... has the team’s interests at heart when he or she acts.	◎	◎	◎	◎	◎	◎	◎
... makes people feel as if they are part of the same team.	◎	◎	◎	◎	◎	◎	◎
... creates a sense of cohesion within the team.	◎	◎	◎	◎	◎	◎	◎
... develops an understanding of what it means to be a member of the team.	◎	◎	◎	◎	◎	◎	◎
... shapes members’ perceptions of the team’s values and ideals.	◎	◎	◎	◎	◎	◎	◎
... devises activities that bring the team together.	◎	◎	◎	◎	◎	◎	◎
... arranges events that help the team function effectively.	◎	◎	◎	◎	◎	◎	◎
... creates structures that are useful for team members.	◎	◎	◎	◎	◎	◎	◎

Arabic

في القادم، نود منك أن تفكر في فريقك ومدير فريقك . يرجى التركيز على نفس الفريق والمدير

مديري المباشر...

	1<br>لا أوافق بشدة	2	3	4	5	6	7<br>أوافق بشدة
ن مديري المباشر يجسد ما تمثله مجموعة العمل	◎	◎	◎	◎	◎	◎	◎
إن مديري المباشر يمثل أعضاء الفريق في العمل	◎	◎	◎	◎	◎	◎	◎
إن مديري المباشر مثل في فريق العمل	◎	◎	◎	◎	◎	◎	◎
إن مديري المباشر يوضح ما يعنيه أن تكون عضوا في فريق العمل	◎	◎	◎	◎	◎	◎	◎
إن مديري المباشر يعزز مصالح أعضاء الفريق في العمل	◎	◎	◎	◎	◎	◎	◎
إن مديري المباشر يتصرف مثل بطل في فريق العمل	◎	◎	◎	◎	◎	◎	◎
إن مديري المباشر يدافع على فريق العمل	◎	◎	◎	◎	◎	◎	◎
إن مديري المباشر يهتم بمصالح الفريق عندما يتصرف	◎	◎	◎	◎	◎	◎	◎
إن مديري المباشر يضع الناس في وضعية سهلة كما لو كانوا جزأ من نفس المجموعة	◎	◎	◎	◎	◎	◎	◎
إن مديري المباشر يخلق شعور التماسك في فريق العمل	◎	◎	◎	◎	◎	◎	◎
إن مديري المباشر يطور رؤية عامة لما يعنيه ان يكون الإ نسان عضوا من فريق في عمله	◎	◎	◎	◎	◎	◎	◎
إن مديري المباشر يبني المثل و القيم التي يشاركها أعضاء الفريق	◎	◎	◎	◎	◎	◎	◎
إن مديري المباشر يطور الأنشطة التي تلحم الفريق	◎	◎	◎	◎	◎	◎	◎
إن مديري المباشر ينظم الأحداث لكي تساعد الفريق على العمل بكفاءة	◎	◎	◎	◎	◎	◎	◎
إن مديري المباشر يخلق هياكل مفيدة لأعضاء فريق العمل	◎	◎	◎	◎	◎	◎	◎

Bosnian

U nastavku želimo da razmislite o Vašem timu i vođi tima (Vaš nadređeni vođa). Molimo Vas da se fokusirate na isti tim ljudi i na istog vođu tima do kraja ovog upitnika.

Moj neposredni vođa…

	U potpunosti se ne slažem 1	2	3	4	5	6	U potpunosti se slažem 7
... utjelovljuje ono za sta se grupa zalaže.	◎	◎	◎	◎	◎	◎	◎
… je reprezentativan predstavnik grupe.	◎	◎	◎	◎	◎	◎	◎
... je uzorni član grupe.	◎	◎	◎	◎	◎	◎	◎
... predstavlja uzor šta znači biti član grupe.	◎	◎	◎	◎	◎	◎	◎
... zalaže se za interese članova grupe.	◎	◎	◎	◎	◎	◎	◎
... borac je za grupu.	◎	◎	◎	◎	◎	◎	◎
... se zalaže za grupu.	◎	◎	◎	◎	◎	◎	◎
... ima interese tima u srcu kada djeluje.	◎	◎	◎	◎	◎	◎	◎
... odaje zaposlenicima osjećaj da su dio grupe.	◎	◎	◎	◎	◎	◎	◎
... kreira osjećaj jedinstva unutar grupe.	◎	◎	◎	◎	◎	◎	◎
... razvija osjećaj razumijevanja kako biti član grupe.	◎	◎	◎	◎	◎	◎	◎
... oblikuje percepciju zaposlenika o grupnim vrijednostima i idealima.	◎	◎	◎	◎	◎	◎	◎
... osmišljava aktivnosti koje spajaju grupu.	◎	◎	◎	◎	◎	◎	◎
... organizira aktivnosti koje pomažu grupi da funkcioniše efektivno.	◎	◎	◎	◎	◎	◎	◎
... stvara procedure koje su korisne članovima tima.	◎	◎	◎	◎	◎	◎	◎

Brazilian Portuguese

Agora gostaríamos que pensasse sobre o seu líder e a sua equipe (por ex. o seu supervisor direto) .
Por favor, ao longo do questionário pense somente na mesma equipe e no mesmo supervisor.

O líder da minha equipe ...

	Discordo completamente 1	2	3	4	5	6	Concordo completamente 7
... personifica aquilo o que a equipe acredita.	◎	◎	◎	◎	◎	◎	◎
… é um bom representante dos membros da equipe.	◎	◎	◎	◎	◎	◎	◎
... é um elemento modelo da equipe.	◎	◎	◎	◎	◎	◎	◎
... exemplifica o que significa ser membro da equipe.	◎	◎	◎	◎	◎	◎	◎
... promove os interesses dos membros da equipe.	◎	◎	◎	◎	◎	◎	◎
... atua como um defensor da equipe.	◎	◎	◎	◎	◎	◎	◎
... defende a equipe.	◎	◎	◎	◎	◎	◎	◎
... tem os interesses da equipe em mente quando age.	◎	◎	◎	◎	◎	◎	◎
... faz com que as pessoas se sintam parte da mesma equipe.	◎	◎	◎	◎	◎	◎	◎
... cria um senso de coesão dentro da equipe.	◎	◎	◎	◎	◎	◎	◎
... desenvolve um entendimento acerca do que significa ser membro da equipe.	◎	◎	◎	◎	◎	◎	◎
... modela as percepções dos membros sobre os valores e os ideais da equipe.	◎	◎	◎	◎	◎	◎	◎
... planeja atividades que une a equipe.	◎	◎	◎	◎	◎	◎	◎
... organiza eventos que ajuda a equipe a funcionar de forma mais eficaz.	◎	◎	◎	◎	◎	◎	◎
... cria estruturas que são úteis para os membros da equipe.	◎	◎	◎	◎	◎	◎	◎

Chinese

领导力评估问卷

下面，请您想想您所在的团队和您的团队领导（也就是您的直接上司）。
在整个问卷调查的过程中，请始终关注同一个团队和相应的领导者。

	完全不同意 1	2	3	4	5	6	完全同意 7
我的直接上司身上体现了团队的文化与价值观。	◎	◎	◎	◎	◎	◎	◎
我的直接上司是团队成员的典型代表。	◎	◎	◎	◎	◎	◎	◎
我的直接上司是团队的一名模范成员。	◎	◎	◎	◎	◎	◎	◎
我的直接上司以身作则，率先垂范，做好团队一员。	◎	◎	◎	◎	◎	◎	◎
我的直接上司能够增进团队成员的利益。	◎	◎	◎	◎	◎	◎	◎
我的直接上司充当团队的捍卫者。	◎	◎	◎	◎	◎	◎	◎
我的直接上司拥护团队。	◎	◎	◎	◎	◎	◎	◎
我的直接上司时刻将团队利益放在心中。	◎	◎	◎	◎	◎	◎	◎
我的直接上司能让大家觉得每个成员都是团队的一份子	◎	◎	◎	◎	◎	◎	◎
我的直接上司在团队内营造凝聚力。	◎	◎	◎	◎	◎	◎	◎
我的直接上司能提高大家对于成为团队成员的意义的理解	◎	◎	◎	◎	◎	◎	◎
我的直接上司塑造了成员的团队价值观和团队理想	◎	◎	◎	◎	◎	◎	◎
我的直接上司能够设计出有利于团队团结的活动	◎	◎	◎	◎	◎	◎	◎
我的直接上司能安排有助于团队有效运作的活动	◎	◎	◎	◎	◎	◎	◎

我的直接上司创建了有益于成员的团队结构。	◎	◎	◎	◎	◎	◎	◎

Dutch

Hieronder willen we dat u denkt aan uw team en leidinggevende (d.w.z. uw directe leidinggevende). Richt u zich gedurende de gehele enquête alstublieft steeds op hetzelfde team en dezelfde bijbehorende leidinggevende.

De leidinggevende die ik beoordeel …

	elemaal niet akkoord<br>1	2	3	4	5	6	Helemaal akkoord<br>7
... belichaamt waar ons team voor staat	◎	◎	◎	◎	◎	◎	◎
…is representatief voor de leden van mijn team	◎	◎	◎	◎	◎	◎	◎
…is een goed voorbeeld van een lid van ons team	◎	◎	◎	◎	◎	◎	◎
…maakt duidelijk wat het betekent om lid te zijn van dit team.	◎	◎	◎	◎	◎	◎	◎
…behartigt de belangen van de leden van mijn team	◎	◎	◎	◎	◎	◎	◎
... gedraagt zich als een voorvechter voor het team	◎	◎	◎	◎	◎	◎	◎
... komt op voor het team.	◎	◎	◎	◎	◎	◎	◎
…heeft de belangen van het team voor ogen wanneer hij/zij iets doet.	◎	◎	◎	◎	◎	◎	◎
…geeft mensen het gevoel dat ze deel uitmaken van één en hetzelfde team	◎	◎	◎	◎	◎	◎	◎
…creëert een gevoel van saamhorigheid binnen het team.	◎	◎	◎	◎	◎	◎	◎
…creëert een beeld van wat het betekent om deel uit te maken van het team	◎	◎	◎	◎	◎	◎	◎
… vormt leden hun percepties over de waarden en idealen van het	◎	◎	◎	◎	◎	◎	◎
… bedenkt activiteiten die het team bij elkaar brengt.	◎	◎	◎	◎	◎	◎	◎

…organiseert activiteiten die het team helpen effectiever te functioneren.	◎	◎	◎	◎	◎	◎	◎
…creëert omstandigheden die bevorderlijk zijn voor het team.	◎	◎	◎	◎	◎	◎	◎

Dutch – Belgium

Hieronder willen we dat u denkt aan uw team en leidinggevende (d.w.z. uw directe leidinggevende). Richt u zich gedurende de gehele enquête alstublieft steeds op hetzelfde team en dezelfde bijbehorende leidinggevende.

De leidinggevende die ik beoordeel …

	Helemaal niet akkoord **1**	**2**	**3**	**4**	**5**	**6**	Helemaal akkoord **7**
...belichaamt waar ons team voor staat	◎	◎	◎	◎	◎	◎	◎
…is representatief voor de leden van mijn team	◎	◎	◎	◎	◎	◎	◎
…is een model-lid van ons team.	◎	◎	◎	◎	◎	◎	◎
…maakt duidelijk wat het betekent om lid te zijn van dit team.	◎	◎	◎	◎	◎	◎	◎
…behartigt de belangen van de leden van mijn team	◎	◎	◎	◎	◎	◎	◎
...gedraagt zich als een voorvechter van het team	◎	◎	◎	◎	◎	◎	◎
...komt op voor het team.	◎	◎	◎	◎	◎	◎	◎
…heeft de belangen van het team voor ogen wanneer hij/zij iets doet.	◎	◎	◎	◎	◎	◎	◎
…geeft mensen het gevoel dat ze deel uitmaken van één en hetzelfde team	◎	◎	◎	◎	◎	◎	◎
…creëert een gevoel van saamhorigheid binnen het team.	◎	◎	◎	◎	◎	◎	◎

…creëert een beeld van wat het betekent om deel uit te maken van het team	◎	◎	◎	◎	◎	◎	◎
…vormt de percepties van teamleden over de waarden en	◎	◎	◎	◎	◎	◎	◎
…bedenkt activiteiten die het team bij elkaar brengen.	◎	◎	◎	◎	◎	◎	◎
…organiseert activiteiten die het team helpen effectiever te functioneren.	◎	◎	◎	◎	◎	◎	◎
…creëert omstandigheden die bevorderlijk zijn voor het team.	◎	◎	◎	◎	◎	◎	◎

Filipino

Isipin mo ang iyong team o mga katrabaho at ang iyong team leader (ang iyong immediate supervisor o boss). Mag-focus sa parehong mga kasamahan at leader habang sinasagutan ang survey.

Ang aking team leader sa trabaho ay...

	**Hinding-hindi sumasang-** 1	2	3	4	5	6	**Lubos na sumasang-ayon** 7
...kumakatawan sa kung ano ang	◎	◎	◎	◎	◎	◎	◎
...kumakatawan sa mga miyembro ng aming team.	◎	◎	◎	◎	◎	◎	◎
...mabuting halimbawa sa aming team.	◎	◎	◎	◎	◎	◎	◎
...isang halimbawa kung ano ang ibig sabihin ng pagiging miyembro ng	◎	◎	◎	◎	◎	◎	◎
...inuuna ang kapakanan ng mga miyembro ng aming team.	◎	◎	◎	◎	◎	◎	◎
...kumikilos bilang isang champion para sa	◎	◎	◎	◎	◎	◎	◎
...naninindigan para sa aming team.	◎	◎	◎	◎	◎	◎	◎
...nasa puso ang kapakanan ng team kapag siya ay kikilos.	◎	◎	◎	◎	◎	◎	◎
...ipinararamdam sa mga tao na sila ay bahagi ng parehong team.	◎	◎	◎	◎	◎	◎	◎

...gumagawa ng pagkakaisa sa loob ng team.	◎	◎	◎	◎	◎	◎	◎
...Nakagagawa ng pagkakaunawaan sa kung ano ang ibig sabihin ng pagiging	◎	◎	◎	◎	◎	◎	◎
...hinuhulma ang persepsyon ng team tungkol sa kanilang pagpapahalaga at	◎	◎	◎	◎	◎	◎	◎
...gumagawa ng mga aktibidad na maglalapit sa mga miyembro.	◎	◎	◎	◎	◎	◎	◎
...Nagsasagawa ng mga events na nakatutulong sa team na mag-function	◎	◎	◎	◎	◎	◎	◎
...gumagawa ng structures na kapaki-pakinabang para sa mga miyembro ng	◎	◎	◎	◎	◎	◎	◎

French

Ci-dessous, nous aimerions que vous pensiez à votre équipe de travail et à votre responsable (supérieur direct). Merci de vous concentrer sur la même équipe et le/la même responsable tout au long de l'étude.

Mon supérieur direct…

	**Complètement en désaccord** 1	2	3	4	5	6	**Complètement d'accord** 7
... incarne ce que le groupe représente.	◎	◎	◎	◎	◎	◎	◎
... est représentatif des membres de l'équipe.	◎	◎	◎	◎	◎	◎	◎
... est un modèle dans l'équipe.	◎	◎	◎	◎	◎	◎	◎
... illustre ce que signifie être membre de l'équipe.	◎	◎	◎	◎	◎	◎	◎
... promeut les intérêts des membres de l'équipe.	◎	◎	◎	◎	◎	◎	◎
... agit comme un champion pour l'équipe.	◎	◎	◎	◎	◎	◎	◎
... défend l'équipe.	◎	◎	◎	◎	◎	◎	◎
... a à cœur les intérêts de l'équipe lorsqu'il/elle agit.	◎	◎	◎	◎	◎	◎	◎

... met les gens à l'aise comme s'ils faisaient partie du même groupe.	◎	◎	◎	◎	◎	◎	◎
... créé de la cohésion au sein de l'équipe.	◎	◎	◎	◎	◎	◎	◎
... développe une vision de ce que signifie être un membre de l'équipe.	◎	◎	◎	◎	◎	◎	◎
... modèle la perception des valeurs et idéaux du groupe par ses membres.	◎	◎	◎	◎	◎	◎	◎
... met en place des activités qui soudent l'équipe.	◎	◎	◎	◎	◎	◎	◎
... organise des évènements qui aident l'équipe à fonctionner efficacement.	◎	◎	◎	◎	◎	◎	◎
... créé des structures qui sont utiles pour les membres du groupe.	◎	◎	◎	◎	◎	◎	◎

German

Im Folgenden möchten wir Sie bitten, möglichst genau über ihr eigenes Team und die dazugehörige Führungskraft (d.h., ihren eigenen Vorgesetzten) nachzudenken. Bitte beziehen Sie sich bei Ihren Aussagen über den ganzen Fragebogen hinweg immer auf dasselbe Team und die dazugehörige Führungskraft.

Die Führungskraft, die ich einschätze, …

	**stimme überhaupt nicht zu** **1**	**2**	**3**	**4**	**5**	**6**	**stimme voll zu** **7**
... verkörpert, wofür das Team steht.	◎	◎	◎	◎	◎	◎	◎
... ist ein typischer Vertreter des Teams.	◎	◎	◎	◎	◎	◎	◎
... ist ein gutes Beispiel eines Teammitgliedes.	◎	◎	◎	◎	◎	◎	◎
... lebt vor, was es bedeutet, ein Mitglied des Teams zu sein.	◎	◎	◎	◎	◎	◎	◎
... fördert die Interessen der Teammitglieder.	◎	◎	◎	◎	◎	◎	◎
... ist ein Verfechter der Interessen des Teams.	◎	◎	◎	◎	◎	◎	◎
... setzt sich für das Team ein.	◎	◎	◎	◎	◎	◎	◎

... hat bei dem, was sie tut, stets die Interessen des Teams im Blick.	◎	◎	◎	◎	◎	◎	◎
... gibt den Mitarbeitern das Gefühl, dass alle zum selben Team gehören.	◎	◎	◎	◎	◎	◎	◎
... schafft ein Gefühl des Zusammenhalts im Team.	◎	◎	◎	◎	◎	◎	◎
... schafft ein Verständnis davon, was es heißt, ein Mitglied des Teams zu sein.	◎	◎	◎	◎	◎	◎	◎
... formt die Wahrnehmung der Werte und Ideale des Teams durch die Teammitglieder.	◎	◎	◎	◎	◎	◎	◎
... denkt sich Aktionen aus, die das Team zusammenbringen.	◎	◎	◎	◎	◎	◎	◎
... organisiert Events, die dem Team helfen, effektiv zusammenzuarbeiten.	◎	◎	◎	◎	◎	◎	◎
... schafft Strukturen, die für die Teammitglieder nützlich sind.	◎	◎	◎	◎	◎	◎	◎

Greek

Θέλουμε να σκεφτείς για την ομάδα εργασίας σου και τον ηγέτη της και να επικεντρωθείς στην ίδια ομάδα και στον ίδιο ηγέτη/προϊστάμενο καθ’ όλη την έκταση της έρευνας.

Ο ηγέτης που αναφέρομαι…

	Απόλυτη διαφωνία 1	2	3	4	5	6	Απόλυτη συμφωνία 7
...ενσαρκώνει αυτό που είναι η ομάδα.	◎	◎	◎	◎	◎	◎	◎
…αντιπροσωπεύει τα μέλη της ομάδας.	◎	◎	◎	◎	◎	◎	◎
...είναι υπόδειγμα μέλους για την ομάδα.	◎	◎	◎	◎	◎	◎	◎
...είναι παράδειγμα για το τι σημαίνει μέλος της ομάδας.	◎	◎	◎	◎	◎	◎	◎
...προωθεί τα συμφέροντα των μελών της ομάδας.	◎	◎	◎	◎	◎	◎	◎
...προασπίζεται την ομάδα.	◎	◎	◎	◎	◎	◎	◎

...υποστηρίζει την ομάδα.	◎	◎	◎	◎	◎	◎	◎
…αντιμετωπίζει σαν αν είναι δικά του τα συμφέροντα της ομάδας.	◎	◎	◎	◎	◎	◎	◎
...μας κάνει να αισθανόμαστε μέλη της ίδιας ομάδας.	◎	◎	◎	◎	◎	◎	◎
...δημιουργεί αίσθημα συνοχής στην ομάδα.	◎	◎	◎	◎	◎	◎	◎
...μας κάνει να κατανοούμε τι σημαίνει μέλος της ομάδας.	◎	◎	◎	◎	◎	◎	◎
...διαμορφώνει τις αντιλήψεις των μελών για τις αξίες και τα ιδεώδη της ομάδας.	◎	◎	◎	◎	◎	◎	◎
...οργανώνει εκδηλώσεις για να φέρει κοντά την ομάδα.	◎	◎	◎	◎	◎	◎	◎
...οργανώνει εκδηλώσεις που βοηθούν την ομάδα να λειτουργεί αποτελεσματικά.	◎	◎	◎	◎	◎	◎	◎
...διαμορφώνει δομές χρήσιμες για τα μέλη της ομάδας.	◎	◎	◎	◎	◎	◎	◎

Hebrew

הפריטים הבאים מתייחסים לצוות שלך ולמנהל/ת שלך במקום העבודה. אנא התרכז/י באותו צוות ובאותו/אותה מנהל/ת בעבודתך לאורך כל השאלון.

המנהל/ת שלי...

	כלל לא מסכים/ה 1	2	3	4	5	6	מסכים/ה לחלוטין 7
מגלם/ת את מה שהקבוצה מייצגת.	◎	◎	◎	◎	◎	◎	◎
מייצג/ת את חברי הקבוצה.	◎	◎	◎	◎	◎	◎	◎
הינו/הינה דמות מופת (מודל) של חברי הקבוצה.	◎	◎	◎	◎	◎	◎	◎
מדגים/ה את המשמעות של להיות חבר/ה בקבוצה.	◎	◎	◎	◎	◎	◎	◎
מקדם/ת את האינטרסים של חברי הקבוצה.	◎	◎	◎	◎	◎	◎	◎
מגן/ה על חברי הקבוצה.	◎	◎	◎	◎	◎	◎	◎
מצדד/ת ותומך/ת בקבוצה.	◎	◎	◎	◎	◎	◎	◎

נוצר/ת בליבו/בליבה את האינטרסים של הקבוצה כאשר הוא/היא פועל/ת.	◎	◎	◎	◎	◎	◎	◎
גורם/ת לאנשים להרגיש חלק מאותה הקבוצה.	◎	◎	◎	◎	◎	◎	◎
יוצר/ת תחושת לכידות בתוך הקבוצה.	◎	◎	◎	◎	◎	◎	◎
מפתח/ת הבנה לגבי המשמעות של להיות חבר/ה בקבוצה.	◎	◎	◎	◎	◎	◎	◎
מעצב/ת את תפיסות החברים בנוגע לאידאלים והערכים של הקבוצה.	◎	◎	◎	◎	◎	◎	◎
מתכנן/ת פעילויות שמקרבות בין חברי הקבוצה.	◎	◎	◎	◎	◎	◎	◎
מארגן/ת אירועים שמסייעים לקבוצה לתפקד ביעילות.	◎	◎	◎	◎	◎	◎	◎
יוצר/ת תשתיות ומבנים שימושיים לחברי הקבוצה.	◎	◎	◎	◎	◎	◎	◎

Italian

Qui di seguito, le chiediamo di pensare al suo gruppo di lavoro e al suo responsabile (es. il suo diretto supervisore). **<u>La preghiamo di concentrarsi sempre sullo stesso gruppo di lavoro e</u> <u>sullo stesso responsabile per il resto del questionario</u>.**

Il responsabile del mio gruppo di lavoro …

	COMPLETA MENTE IN DISACCOR 1	2	3	4	5	6	COMPLETAME NTE [illegible] 7
… incarna ciò che il gruppo rappresenta	◎	◎	◎	◎	◎	◎	◎
… è rappresentativo dei membri del gruppo	◎	◎	◎	◎	◎	◎	◎
… è un modello per il gruppo	◎	◎	◎	◎	◎	◎	◎
… è un esempio di ciò che significa essere un membro del gruppo	◎	◎	◎	◎	◎	◎	◎
… promuove gli interessi dei membri del gruppo	◎	◎	◎	◎	◎	◎	◎
… agisce come sostenitore del gruppo	◎	◎	◎	◎	◎	◎	◎
… prende le difese del gruppo	◎	◎	◎	◎	◎	◎	◎

… quando agisce ha a cuore gli interessi del gruppo	◎	◎	◎	◎	◎	◎	◎
… fa sentire le persone come se fossero parte dello stesso gruppo	◎	◎	◎	◎	◎	◎	◎
… crea un senso di coesione all'interno del gruppo	◎	◎	◎	◎	◎	◎	◎
… sviluppa il significato di ciò che vuol dire far parte del gruppo	◎	◎	◎	◎	◎	◎	◎
… dà forma ai valori e agli ideali del gruppo	◎	◎	◎	◎	◎	◎	◎
… progetta attività che tengono insieme il gruppo	◎	◎	◎	◎	◎	◎	◎
… organizza eventi che aiutano il gruppo a funzionare efficacemente	◎	◎	◎	◎	◎	◎	◎
… fornisce risorse strutturali utili per i membri del gruppo	◎	◎	◎	◎	◎	◎	◎

Japanese

私のリーダーは …

	全くそう思わない 1	2	3	4	5	6	完全にそう思う 7
このチームが象徴するものを体現し	◎	◎	◎	◎	◎	◎	◎
チームのメンバーを代表している	◎	◎	◎	◎	◎	◎	◎
チームでは見本となる人物である	◎	◎	◎	◎	◎	◎	◎
このチームの一員であるということは何を意味するかの見本となっている。	◎	◎	◎	◎	◎	◎	◎
チームのメンバーたちの利益を促進しようとしている	◎	◎	◎	◎	◎	◎	◎
チームの擁護者として活動している	◎	◎	◎	◎	◎	◎	◎
チームのために立ち上がる	◎	◎	◎	◎	◎	◎	◎
彼／彼女が活動するときは、チームの利害をわきまえている	◎	◎	◎	◎	◎	◎	◎
人々を、同じチームの一員だと感じさせるようにする	◎	◎	◎	◎	◎	◎	◎

チーム内で団結意識を生み出す	◎	◎	◎	◎	◎	◎	◎
このチームの一員であることは何を意味するのかについての理解を促進	◎	◎	◎	◎	◎	◎	◎
チームの価値観や理想についての共通認識を形成させる	◎	◎	◎	◎	◎	◎	◎
チームを団結させるための活動を考案する	◎	◎	◎	◎	◎	◎	◎
チームが効果的に機能するようなイベントを企画する	◎	◎	◎	◎	◎	◎	◎
チームのメンバーにとって役に立つチーム内構造を作り出す	◎	◎	◎	◎	◎	◎	◎

Norwegian

Nedenfor vil vi be deg tenke på ditt eget team og din leder. Gjennom hele spørreundersøkelsen ønsker vi at du tar utgangspunkt i det samme teamet og den/de samme lederen/overordnede.

Lederen som jeg vurderer …

	**helt uenig** **1**	**2**	**3**	**4**	**5**	**6**	**helt enig** **7**
… representerer det teamet står for	◎	◎	◎	◎	◎	◎	◎
… er representativ for teamet	◎	◎	◎	◎	◎	◎	◎
… er modell for teamet	◎	◎	◎	◎	◎	◎	◎
… er et foregangseksempel som teammedlem	◎	◎	◎	◎	◎	◎	◎
… fremmer teammed-lemmenes interesser	◎	◎	◎	◎	◎	◎	◎
… forfekter teamets interesser	◎	◎	◎	◎	◎	◎	◎
… taler teamets sak	◎	◎	◎	◎	◎	◎	◎
… har alltid teamets interesser i tankene	◎	◎	◎	◎	◎	◎	◎
… gir medlemmene følelsen av å tilhøre samme team	◎	◎	◎	◎	◎	◎	◎

… skaper følelsen av samhold i teamet	◎	◎	◎	◎	◎	◎	◎
… utvikler forståelse for teammedlemskap	◎	◎	◎	◎	◎	◎	◎
… utvikler teamets forståelse for felles verdier og idealer	◎	◎	◎	◎	◎	◎	◎
… tenker ut aktiviteter som samler team-medlemmene	◎	◎	◎	◎	◎	◎	◎
… gjennomfører tiltak og 'events' slik at teamet fungerer effektivt	◎	◎	◎	◎	◎	◎	◎
… utvikler nyttige teamstrukturer	◎	◎	◎	◎	◎	◎	◎

Polish

Chcielibyśmy, abyś przemyślał/a kilka kwestii dotyczących zespołu, w którym pracujesz, i waszego lidera (np. bezpośredniego przełożonego). Prosimy, abyś w całej ankiecie skupił/a się na tym właśnie zespole i liderze.

Lider mojego zespołu…

	**Zupełnie się nie zgadzam** 1	2	3	4	5	6	**Zgadzam się całkowicie** 7
... uosabia to, co akceptuje zespół	o	o	o	o	o	o	o
… reprezentuje członków zespołu	o	o	o	o	o	o	o
... jest uosobieniem zespołu	o	o	o	o	o	o	o
... jest przykładem tego, co znaczy być członkiem zespołu.	o	o	o	o	o	o	o
... promuje interesy członków zespołu.	o	o	o	o	o	o	o
... działa jak promotor zespołu.	o	o	o	o	o	o	o
... staje w obronie zespołu.	o	o	o	o	o	o	o
... swoimi działaniami pokazuje, że interes zespołu leży mu/jej na sercu.	o	o	o	o	o	o	o

... sprawia, że ludzie mają poczucie, iż są częścią tego samego zespołu.	o	o	o	o	o	o	o
... kreuje poczucie jedności w zespole.	o	o	o	o	o	o	o
... rozwija zrozumienie tego, co znaczy być członkiem zespołu.	o	o	o	o	o	o	o
... kształtuje u członków grupy sposób postrzegania wartości i ideałów zespołu.	o	o	o	o	o	o	o
... wymyśla działania spajające zespół.	o	o	o	o	o	o	o
... aranżuje wydarzenia, które pomagają zespołowi skutecznie działać.	o	o	o	o	o	o	o
... tworzy struktury użyteczne dla członków zespołu.	o	o	o	o	o	o	o

Portuguese

De seguida, gostaríamos que pensasse acerca da sua equipa e do líder da sua equipa (i.e., a sua chefia directa). Por favor foque-se na mesma equipa e correspondente supervisor ao longo do questionário.

O líder da minha equipa …

	**Discordo completamente** **1**	**2**	**3**	**4**	**5**	**6**	**Concordo completamente** **7**
... encarna aquilo que a equipa simboliza.	◎	◎	◎	◎	◎	◎	◎
… é representativo dos membros da equipa .	◎	◎	◎	◎	◎	◎	◎
... é um elemento modelo da equipa .	◎	◎	◎	◎	◎	◎	◎
... exemplifica o que significa ser membro da equipa.	◎	◎	◎	◎	◎	◎	◎
... promove os interesses dos membros da equipa.	◎	◎	◎	◎	◎	◎	◎
... actua como um campeão pela equipa.	◎	◎	◎	◎	◎	◎	◎
... defende a equipa.	◎	◎	◎	◎	◎	◎	◎
... tem os interesses da equipa em mente quando ele ou ela age.	◎	◎	◎	◎	◎	◎	◎

... faz com que as pessoas se sintam parte da mesma equipa.	◎	◎	◎	◎	◎	◎	◎
... cria um sentido de coesão dentro da equipa.	◎	◎	◎	◎	◎	◎	◎
... desenvolve uma compreensão acerca do que significa ser membro da equipa.	◎	◎	◎	◎	◎	◎	◎
... modela as percepções dos membros sobre os valores e os ideais da equipa.	◎	◎	◎	◎	◎	◎	◎
... desenha actividades que unem a equipa.	◎	◎	◎	◎	◎	◎	◎
... organiza eventos que ajudam a equipa a funcionar eficazmente.	◎	◎	◎	◎	◎	◎	◎
... cria estruturas que são úteis para os membros da equipa.	◎	◎	◎	◎	◎	◎	◎

Russian

Отвечая на представленные ниже вопросы, пожалуйста, думайте о вашем коллективе и его *лидере* (то есть о вашем непосредственном руководителе). Весь опрос построен на вашей оценке именно вашего коллектива и вашего руководителя.

Лидер (руководитель) моего коллектива ...

	**Полность ю не согласен**<br>**1**	**2**	**3**	**4**	**5**	**6**	**Согласен полность ю**<br>**7**
... воплощает то, чем является наш коллектив.	☐	☐	☐	☐	☐	☐	☐
... является представителем членов коллектива.	☐	☐	☐	☐	☐	☐	☐
... является образцовым членом коллектива.	☐	☐	☐	☐	☐	☐	☐
... иллюстрирует, что значит быть членом коллектива.	☐	☐	☐	☐	☐	☐	☐

... продвигает интересы членов коллектива.	☐	☐	☐	☐	☐	☐	☐
… готов защищать коллектив.	☐	☐	☐	☐	☐	☐	☐
... готов отстаивать интересы коллектива.	☐	☐	☐	☐	☐	☐	☐
... в своей работе руководствуется интересами коллектива.	☐	☐	☐	☐	☐	☐	☐
... заставляет людей чувствовать, что они являются частью одной команды.	☐	☐	☐	☐	☐	☐	☐
... создает чувство сплоченности в коллективе.	☐	☐	☐	☐	☐	☐	☐
... формирует понимание того, что значит быть членом коллектива.	☐	☐	☐	☐	☐	☐	☐
... формирует восприятие общих ценностей и идеалов среди членов коллектива.	☐	☐	☐	☐	☐	☐	☐
... разрабатывает мероприятия, которые объединяют коллектив.	☐	☐	☐	☐	☐	☐	☐
... организует мероприятия, которые помогают коллективу эффективно функционировать.	☐	☐	☐	☐	☐	☐	☐
... создает структуры, которые полезны для членов коллектива.	☐	☐	☐	☐	☐	☐	☐

Slovene

V sklopu spodaj bi radi, da se osredotočite na vašo delovno enoto in vašega vodjo (neposrednega nadrejenega/nadzornika). Prosimo, da se v celotni raziskavi osredotočate na isto delovno enoto in isto oseb.

Moj neposredni nadrejeni …

	**Sploh se ne strinjam** 1	2	3	4	5	6	**Popolnoma se strinjam** 7
... uteleša to, za kar se zavzema skupina.	o	o	o	o	o	o	o
… je predstavnik članov skupine.	o	o	o	o	o	o	o
... je vzorni član skupine.	o	o	o	o	o	o	o
... ponazarja, kaj pomeni biti član skupine.	o	o	o	o	o	o	o
... se zavzema za interese članov skupine.	o	o	o	o	o	o	o
... deluje kot zagovornik skupine.	o	o	o	o	o	o	o
... se postavi za skupino.	o	o	o	o	o	o	o
... ima pri svojih dejanjih v mislih interese skupine.	o	o	o	o	o	o	o
... daje ljudem občutek, da so del iste skupine.	o	o	o	o	o	o	o
... ustvarja občutek povezanosti znotraj skupine.	o	o	o	o	o	o	o
... razvija razumevanje o tem, kaj pomeni biti član skupine.	o	o	o	o	o	o	o
... oblikuje zaznavo članov o vrednotah in idealih skupine.	o	o	o	o	o	o	o
... načrtuje aktivnosti, ki združujejo skupino.	o	o	o	o	o	o	o
... organizira dogodke, ki pomagajo skupini delovati učinkovito.	o	o	o	o	o	o	o
... ustvarja postopke, ki so uporabni za člane skupine.	o	o	o	o	o	o	o

Spanish

A continuación, nos gustaría que pensases sobre tu equipo de trabajo y la persona que lidera el mismo (esto es, tu supervisor/a directo/a). Por favor, céntrate en el mismo equipo y su correspondiente líder a lo largo de toda la encuesta.

La persona que lidera mi equipo…

	Completamente en desacuerdo 1	2	3	4	5	6	Complemtament de acuerdo 7
... personifica lo que el equipo representa.	◎	◎	◎	◎	◎	◎	◎
… es representativa de los miembros del equipo.	◎	◎	◎	◎	◎	◎	◎
... es un miembro modelo del equipo.	◎	◎	◎	◎	◎	◎	◎
... ejemplifica lo que significa ser miembro del equipo.	◎	◎	◎	◎	◎	◎	◎
... promueve los intereses de los miembros del equipo.	◎	◎	◎	◎	◎	◎	◎
... actúa como un impulsor del equipo.	◎	◎	◎	◎	◎	◎	◎
... defiende al equipo.	◎	◎	◎	◎	◎	◎	◎
... tiene los intereses del equipo presentes cuando actúa.	◎	◎	◎	◎	◎	◎	◎
... hace sentir a la gente que son parte del mismo equipo.	◎	◎	◎	◎	◎	◎	◎
... crea una sensación de cohesión dentro del equipo.	◎	◎	◎	◎	◎	◎	◎
... desarrolla un entendimiento de lo que significa ser un miembro del equipo.	◎	◎	◎	◎	◎	◎	◎
... moldea las percepciones de los miembros sobre los valores e ideales del equipo.	◎	◎	◎	◎	◎	◎	◎
... genera actividades que unen al equipo.	◎	◎	◎	◎	◎	◎	◎

... coordina eventos que ayudan a que el equipo funcione con eficacia.	◎	◎	◎	◎	◎	◎	◎
... crea estructuras que son útiles para los miembros del equipo.	◎	◎	◎	◎	◎	◎	◎

Turkish

Sizden, kendi çalışma ekibinizi/grubunuzu ve bu ekibin/grubun liderini düşünmenizi istiyoruz. Lütfen bütün anket boyunca aynı ekibe ve ekip liderine odaklanın.

Değerlendirdiğim Lider…

	Hiç katılmıyorum 1	2	3	4	5	6	Tamamen katılıyorum 7
... grup neyi savunuyorsa onu temsil eder.	o	o	o	o	o	o	o
… grup üyelerinin temsilcisidir.	o	o	o	o	o	o	o
... grubun örnek üyesidir.	o	o	o	o	o	o	o
... bir grup üyesinin nasıl olması gerektiğine örnektir.	o	o	o	o	o	o	o
... grup üyelerinin çıkarlarını gözetir.	o	o	o	o	o	o	o
... grubun savunucusu olarak davranır.	o	o	o	o	o	o	o
... grubun tarafını tutar.	o	o	o	o	o	o	o
... ne yaparsa yapsın, aklında her zaman grubun çıkarları vardır.	o	o	o	o	o	o	o
... insanlara aynı grubun parçası olduklarını hissettirir.	o	o	o	o	o	o	o
... grup içinde birlik bütünlük hissi yaratır.	o	o	o	o	o	o	o
... grup üyeliğinin ne demek olduğuna ilişkin bir anlayış geliştirir.	o	o	o	o	o	o	o
... , grup değerleri ve idealleriyle ilgili, üyelerin algılarını şekillendirir.	o	o	o	o	o	o	o
... grubu bir araya getirecek etkinlikler bulur.	o	o	o	o	o	o	o

... grubun işlerini etkin şekilde yürütmesine yardımcı olacak faaliyetler düzenler.	o	o	o	o	o	o	o
... grup üyelerinin faydalanacakları düzenlemeler yapar.	o	o	o	o	o	o	o

Urdu

نیچے دیئے گئے سوالات میں ہم چاہتے ہیں کہ آپ اپنی ٹیم اور اپنے لیڈر کے بارے میں سوچیں۔ مہربانی کر کے سارے سروے میں ایک ہی ٹیم اور لیڈرپر توجہ دیں۔

میرا ٹیم لیڈر ---

مکمل اتفاق کرتا ہوں۔ 7	6	5	4	3	2	مکمل اختلاف کرتا ہوں 1	
◎	◎	◎	◎	◎	◎	◎	---ٹیم کے مقاصد کا حصہ ہے۔
◎	◎	◎	◎	◎	◎	◎	--- ٹیم کے اراکین کا نمائندہ ہے۔
◎	◎	◎	◎	◎	◎	◎	--- ٹیم کا مثالی رُکن ہے۔
◎	◎	◎	◎	◎	◎	◎	--- ٹیم کا صیح معانی میں رُکن کی مثال ہے۔
◎	◎	◎	◎	◎	◎	◎	---ارکان کے مفاد کو فروغ دیتا ہے۔
◎	◎	◎	◎	◎	◎	◎	--- ٹیم کے چمپین کا کردار ادا کرتا ہے۔
◎	◎	◎	◎	◎	◎	◎	--- ٹیم کیلئے کھڑا ہوتا ہے۔
◎	◎	◎	◎	◎	◎	◎	---جب کو ئی عمل کرتا ہے تو ٹیم کامفاد مد نظر رکھتاہے۔
◎	◎	◎	◎	◎	◎	◎	---لوگوں کو احساس دیلاتا ہے کہ وہ ایک ہی ٹیم کا حصہ ہیں۔
◎	◎	◎	◎	◎	◎	◎	--- ٹیم میں ہم آہنگی کا احساس پیدا کرتا ہے۔
◎	◎	◎	◎	◎	◎	◎	--- ٹیم کاصیح معانی میں رُکن ہونے کی افہام و تفہم پیدا کرتا ہے۔
◎	◎	◎	◎	◎	◎	◎	---ارکان کے خیالات کو ٹیم کی اقدار اور نظریات کے مطابق ڈھالتا ہے۔
◎	◎	◎	◎	◎	◎	◎	--- ٹیم کے ارکان کو اکٹھاکرنے کیلئےسرگرمیاں واضح کرتا ہے ۔

ـــ ٹیم کی کارکردگی کو بہتر بنانے کیلئے تقریبات کا بندوبست کرتا ہے۔	◎	◎	◎	◎	◎	◎	◎
ـــڈھانچہ تشکیل دیتا ہے جو ٹیم کیلئےکارآمدہو۔	◎	◎	◎	◎	◎	◎	◎
